# Diary of a Mental Health Peer Worker: Findings From a Diary Study Into the Role of Peer Work in a Clinical Mental Health Setting

**DOI:** 10.3389/fpsyt.2020.587656

**Published:** 2020-12-17

**Authors:** Marianne Wyder, Helena Roennfeldt, Stephen Parker, Gabrielle Vilic, Karen McCann, Carolyn Ehrlich, Frances Louise Dark

**Affiliations:** ^1^Metro South Addiction and Mental Health Services, Brisbane, QLD, Australia; ^2^The Hopkins Centre, Menzies Health Institute, Griffith University, Logan, QLD, Australia; ^3^The School of Business, RMIT University, Melbourne, VIC, Australia

**Keywords:** peer work, community care unit, qualitative, mental health, rehabilitation

## Abstract

**Introduction:** The importance of peer support workers in mental health care delivery has been extensively advocated for in mental health policy frameworks. However, there has been limited research examining the implementation of paid peer workers in clinical settings. This study explores the experience of paid peer support workers integrated within a clinically-operated community-based residential rehabilitation service for people diagnosed with a mental health disorder experiencing challenges living independently in the community.

**Methods:** A general inductive approach was taken in the analysis of diaries completed by a newly employed peer workforce. These diaries focussed on what they viewed as significant interactions in fulfilling their role. Composite vignettes were generated to illustrate key themes.

**Findings:** Thirty-six diaries were provided; these reported unplanned and spontaneously occurring interactions. Peer workers emphasized the importance of connecting with people while they were engaging in everyday activities as an opportunity for personal growth of the residents. The diaries also focussed on the peer workers' ability to connect and establish trust by sharing similar experiences with residents or family members. Peer workers also believed that they brought a different perspective than clinical staff and were able to refocus attention from clinical diagnoses and symptoms to other aspects of the resident's lives.

**Discussion:** Peer support workers described their work as flexible, responsive, and adaptable to the resident's needs. They believed that their roles brought a different lens to interactions on the unit and fostered a more inclusive and personal way of working for the team.

**Conclusion:** To ensure that peer workers can engage authentically with residents and family members, it is critical that the role and principles of peer work are valued and understood by all.

## Introduction

Peer support workers are an important and expanding component of the mental health workforce. Peer support workers are people who are employed in government and non-government services, peer operated services and clinical settings on the basis of their lived experiences with mental health distress (1, 2). Peer support work differs from traditional mental health roles in the emphasis on using one's lived experience of mental health issues and recovery to support other's experiencing similar concerns (3, 4). Important aspects of peer support worker roles can include eliciting and promoting the strengths of consumers; supporting self-determination; and advocating to reduce discrimination, leading to improved mental health (5).

The employment of peer workers reflects wider policy reform that recognizes recovery as foundational to mental health service delivery (6). Peer roles exemplify the possibility of recovery for people experiencing mental health distress (3, 7). Understanding recovery for people affected by mental health challenges requires a holistic approach with emphasis on principles such as hope, autonomy, informed choice, social connection, and the strengths of the individual (8, 9).

Between 2012 and 2015 Community Care Units (CCUs), were introduced at the Metro South Addiction and Mental Health services in Queensland, Australia. CCUs were established in Australia in the 1990s as an alternative to long term hospitalization and institutionalization (10). Core features of the CCU model include cluster housing in a community setting combined with the onsite availability of mental health professionals. The MSAMHS CCUs emphasize rehabilitation, working with the residents based on their goals, priorities, and preferences. Foci of care are living skills development (e.g., budgeting, cooking, and cleaning) and community integration (e.g., interpersonal effectiveness, social problem solving, and citizenship) (11). Therapeutic interventions available on site include cognitive behavior therapy, cognitive remediation, and social cognition and interaction training (12).

The peer workforce in two CCU's was envisioned to be a distinct speciality. The role did not encompass clinical care but focussed on using lived experience to help engage residents with a focus on relationship and community inclusion (13). It was envisioned that the peer workforce, under supervision from senior peer workers, would iteratively co-design their roles over time. This was an attempt to mitigate the power imbalance between the mental health service employer and employee and to try to avoid distorting the unique value of the peer workforce by the “contrived and constrained world that is mental health services” (14).

An “integrated staffing model” was adopted in two CCUs. Peer support workers (PSWs) comprised the majority of roles within the multi-disciplinary team (15). A goal of the integrated staffing model was to integrate peer support into a multidisciplinary team to enhance recovery-oriented practice. Several studies provide evidence of the effectiveness of peer work in producing improved outcomes for people accessing mental health services, including facilitating engagement, promoting hope, increasing self-management, reduced hospitalizations, increased satisfaction with services (16–20). Peer support workers also experience improved self-esteem, confidence, employability, and recovery (21–23). The role is not without challenges, including role confusion and lack of role credibility. Difficulties defining and maintaining peer roles can be complicated within clinical settings where tension between recovery and the medical model may be more pronounced (23–26).

While there is now a growing body of research into peer support work (20) there is still limited information on what makes the roles effective from the perspective of the peer support workers (27). To gain a deeper understanding of how peer support workers developed and conceptualized their roles at these early stages, the CCU peer support workers were asked to document and reflect on what they considered to be significant interactions. In this article, we report on the qualitative analysis of these diaries.

## Methodology

### Data Collection Methods

Personal reflections, if documented in proximity to significant events, can be tools to capture the participant's thoughts and feelings of events as they happened (28). While written data is often considered less rich compared to face-to-face interviewing, it has been noted that written answers are often more focussed, condensed, and self-reflective when compared to oral accounts (29). In some ways, written accounts also produce data which can be easier to analyse as people who are interviewed can easily lose their train of thought, be unprepared for certain questions, or be interrupted (30). Furthermore, written responses allow for contemporaneous data collection and descriptions of events viewed by the participants as most significant, without imposing the filter of an interviewer.

To capture early experiences of the newly formed peer workforce, peer support workers were provided with a template to document and reflect on what they considered to be significant interactions. The template was developed by the research team and was intended to be a prompt for the peer support worker to write about their experiences. The questions were open ended and included references to positive and or negative experiences. Peer support workers were invited to write about experiences that they considered important. The questions on this template asked peer workers to: (1) provide a brief description of the interaction; (2) describe what they believed had been helpful (or not) in that interaction; and (3) why they believed that this interaction was significant. Peer support workers were asked to record these interactions in ways that would not allow the resident, or the peer support worker involved to be identified. The diaries were collected over a period of 5 months. It was up to discretion of the peer support worker to fill in the diaries. They were invited to share these diaries anonymously with the research team at the end of the data collection period. A total of 36 diaries were shared for this project. Ethical approval for the analysis of these transcripts for the purposes of this project was received from the relevant ethics committee.

### Data Analysis

A general inductive approach was used to analyse the data. This approach establishes clear links between the research objectives and the summary findings derived from the raw data which then allows the development of a model or theory about the underlying structure of experiences as evidenced in the raw data (31). The data was analyzed in two stages. Stage one involved initial coding of the diaries; all diaries were read, and individual codes were developed. These codes were initially very broad. The codes were then regrouped into themes. The initial coding framework and themes were developed by MW and HR and refined by the research team. This refined coding framework was then re-applied to the full dataset. The diaries were read by MW, HR, CE, KM, and GV. HR identifies as a consumer researcher. The data was managed in ATLAS TI. As there were only a small number of peer workers and residents in the CCUs and to ensure people were not identifiable, themes and diaries were combined into composite cases and case vignettes. Diary entries that are used as examples in this article are based on these composite cases and vignettes, and names given are pseudonyms.

## Results

A total of 36 diaries were provided; 31 reported on interactions with residents and five reported on interactions with carers. In all the diaries, peer support workers predominantly used everyday language to describe various interactions with residents. All diaries reported on unplanned interactions that occurred spontaneously. Three themes were identified, namely: (1) Having time and space to engage with residents; (2) Connecting and sharing similar experiences; and, (3) Providing a peer perspective. Table 1 provides an overview of the overarching and subthemes as well as the frequency these occurred.

**Table 1:** Frequency of themes and subthemes identified.

**Themes and Subthemes**	***n* (%)**
**Time and space**	
Using everyday experiences as opportunities for growth	9 (25)
Learning new skills	6 (18)
**Connecting and sharing experiences**	
Trust to have hard discussions	17 (47)
Sharing similar experiences	16 (44)
Being understood	9 (25)
Not alone	7 (19)
Giving hope	6 (17)
Seeing potential	2 (6)
**Peer perspective**	
Importance of non-clinical interventions	14 (39)
Inclusion	3 (8)
bridge between clinical and lived experience	2 (6)
Enforcing clinical priorities	1 (3)

### Having Time and Space to Connect With Residents

This theme was divided into using everyday experiences as opportunities for growth and learning new skills. A quarter of the diaries described the importance of being able to spend time with people and engage around everyday experiences as opportunities for growth. These activities were generally unscheduled and opportunistic and, at face value, without rehabilitative purpose. Most of these interactions occurred when the peer support workers were with residents in their independent living units. Examples of times the peer support workers connected with residents while engaging in other activities included: “learning to crochet,” “playing pool,” “learning to play an instrument,” and “learning social skills.” In these interactions, peer support workers offered support and encouragement when things didn't go according to plan, as well as, reassurance that feelings of frustration and disappointment were common experiences. The peer support workers believed that residents' abilities to be persistent as well as learning to deal with frustration were important outcomes of these interactions. Other diaries highlighted that participants viewed their support as enabling residents to engage in every day social activities that would ordinarily make them uncomfortable. These interactions were described as allowing residents to develop new coping and social skills as well as becoming more adaptable.

#### Case Vignette: Sarah

Jane, the peer support worker, worked with Sarah to feel less anxious being around people. Sarah had always wanted to learn an instrument but was reluctant to attend the music group. Over a few weeks, Jane spent a lot of time with Sarah to help her attend the group. With a lot of encouragement and Jane's presence, Sarah attended the group. While Sarah, did not want to participate in the group, she enjoyed it. Jane checked in with her afterwards and focussed on her strengths and the positives of her progress. Over the weeks, she was able to attend the group and participate.

“*Sarah was very reluctant to attend stating that she “couldn't possibly go as this is just not for me.”. She came to the group and was quick to state that she wasn't willing to participate but would be happy to watch. I followed up with her to congratulate her on being able to stay throughout the group. Sarah's self-confidence improved as she proved to herself that she was able to attend…*”

### Connecting and Sharing Similar Experiences

Over half the diaries described strategies peer support workers used to connect and establish the trust needed to have difficult discussions. This was most often achieved by sharing with residents (or family members) that they had similar experiences and how they had dealt with these. Peer support workers believed that by sharing that they had similar experiences, a safe space was created for residents to talk about sensitive topics. This allowed residents to talk openly about their struggles and to have meaningful discussions about topics of importance to them. Peer support workers described that, as a result, the residents' shame was lessened because they felt less judged. These sensitive topics included how depression had impacted their lives, fear of being tempted back into using drugs, dealing with family conflict, losing faith, and suicidal thinking. Peer support workers viewed these conversations as different to those residents would have with clinical staff members, in that sharing common experiences showed residents that they were not alone with their challenges; that there are people that understand what they are going through. Peer workers believed that this sharing of experiences was a key strength of their role that allowed them implicitly to share hope that things would improve for residents.

#### Case Vignette: John

John had been experiencing increased anxiety and feeling low. Katelyn, the peer support worker went to see him in his unit to have a chat. He had been experiencing some tension with his family. He and Katelyn spoke about how difficult it can be to manage difficult family members, and Katelyn shared some of her experiences. During the chat, he spoke about his anxiety and wanted some medication to help him deal with the symptoms. Katelyn mentioned some of her own coping strategies with him and provided him with a CDs with music and mediations as well as a CD player to try these out. As a result, he was able to manage his anxiety without taking more medication.

“*I called in to see John in his unit. He told me that he wasn't traveling so well and asked if I would like to have a coffee and chat. We sat and had coffee which helped to normalize our interaction. We were in his unit and he was the host. We engaged in a conversation about his family members. I supported him by listening to his concerns and shared some of my experience. I spoke about what I try to do in situations like this. We talked a lot about coping strategies and John revealed that he was having a great deal of trouble sleeping, averaging only 4 h per night due to stress and anxiety. He was waking up feeling worried and this was compounding an already difficult situation. John felt heard and understood and felt comfortable to chat openly about what depression has meant in the past and the consequences of when it is present in his life. Being a Peer Worker and having the scope both professionally and personally to share my story in a manner that was helpful allowed John to gain and share insight into his own mental health difficulties. I believe that being able to be open and honest about mental illness and really understanding the symptoms and struggles from a lived perspective lets the person know that they are not alone*.”

#### Case Vignette: George

George left the unit and was asked by Jeremy what had been the most helpful during his stay.

*I asked what had been helpful for his recovery at the CCU and he said the Peer Workers as he had not met people with mental health difficulties who work and manage their illness. He said the Peers understood him and were able to help as they knew what he meant when going through the bad times*.

### Provision of the Peer Perspective

The diaries also documented how peer support workers brought a different perspective to clinical staff in the way that they worked. Many of the diaries emphasized the importance of focussing on aspects of people's lives other than clinical diagnoses and symptoms. These included hobbies, cooking, appreciating music or poetry or being able to participate in group activities. These aspects were described as being as important to a person's recovery as were medications and clinical interventions. In their roles, peer support workers believed they were able to shift the focus from a clinical perspective to a lived experience perspective, emphasizing self-management, person directed care, and belief in the person's inherent capacity to overcome adversity. This meant however that, at times, they were at odds with the clinical team and needed to advocate for the wishes of the person to take priority. Peer support workers also felt that they brought a different lens on what may have been seen by clinical staff as symptoms or negative behavior.

#### Case Vignette: Joshua

Many of the clinical staff had been concerned about Joshua's behaviors and they were concerned that he was not working on his recovery.

“*I wasn't comfortable with the idea that Joshua wasn't still focused on his recovery and felt that there was a lack of trust being afforded to him. I was able to get some time alone to talk with Josh and I was finally able to encourage him to come up to the shops with me, which was one of his recovery goals and a seriously anxiety provoking activity for him. In supporting him to do this I felt like I was able to have a considerable breakthrough in what was going on for him because I was able to identify several similar coping mechanisms that we both shared*.”

The peer support workers also described how their roles encouraged inclusion of residents in the CCU. Various diary entries described situations where peer support workers invited residents to participate in activities which in the past were undertaken solely by staff members. These activities included putting up decorations at the CCU, preparing food for staff, or jointly participating in celebrations. Including the residents in these activities appeared to change the dynamics in the CCU and had positive impacts on the residents and staff.

#### Case Vignette: Jack

Fred and Oscar (peer support workers) were preparing for a staff party as there were three birthdays that week. While they were preparing Jack arrived. They asked him if he wanted to help decorate the CCU. While they were decorating, they shared memories of birthdays when they were younger. Once the decorations were done the peer support workers suggested to the staff that the residents join them.

“*Including Jack in a shared activity normalized things for him. This helped make him to feel like he belonged. Participating in the activity also gave him a sense of purpose. A fun relaxed atmosphere was created that allowed Jack to feel comfortable enough to share his childhood memories, and gave him a feeling of safety, acceptance, and self-worth*.”

“*This celebration was the first time that many of the residents had gathered with staff for a social event. People chatted, shared stories, played games, and enjoyed themselves. It was wonderful to observe people (residents/staff) working together with the preparation, the celebration, and the clean-up*.”

At other times, it was challenging for peer support workers to uphold a peer perspective. This was notable in times when resident safety or well-being was considered by clinical staff to be at risk. In one diary entry, the peer support worker was asked to enforce clinical priorities to ensure the person's safety. The peer support worker in this instance described losing the trust of the resident, but the peer support worker reconciled this, stating that “it was more important for the treatment team to maintain the connection.”

## Discussion

This project showcases how peer support workers operationalize their work in a public mental health service and the types of interactions they consider significant in their own words. This study provides important insights into how peer support workers conceptualize their work within an integrated staffing model. Peer support workers strongly emphasize the importance of connecting with people while they are engaging in everyday activities. These interactions were facilitated by the availability of time and a shared space. Connecting with residents through shared engagement in everyday activities was viewed as providing authentic opportunities to support residents deal with their experiences and fears. These interactions were viewed as building relationships and trust. Additionally, the peer support workers viewed self-disclosure of their lived experience as important to establishing trust, as well as reducing shame and isolation. The peer support workers emphasis on shared engagement in everyday experiences aligns with the importance of personal recovery concepts. Personal growth and living a good life may be viewed differently to measures of clinical effectiveness/emphasis on symptom reduction (32). It is likely that the parallel processes of having clinical and peer processes in a service will provide more rounded interventions.

Peer support workers viewed their role as distinct from the clinical staff and believed that their lived experience lens facilitated learning and brought inclusiveness to the CCU environment. The diaries often focused on their role as advocates and change agents within the team. Peer support workers described their roles as reciprocal in building equitable relationships with the residents. Based on these diaries, it is not possible to know if this was experienced in the same way by the residents or staff. However, previous work evaluating the CCUs has suggested that residents as well as staff view the integrated staffing model positively and describe peer support workers as “bridging the gap” between residents and clinical staff by facilitating improved interactions and communication. The peer support workers also played a role in ‘putting things in perspective’ by normalizing through sharing lived experience (11).

This study highlights the specialist skillset peer support workers bring to the role. The diaries suggest that to be able to work effectively within a community-based rehabilitation setting, it is critical to maintain a lived/living experience perspective. To be able to share one's own experiences in a purposeful and meaningful way requires an ability to draw on these experiences with emotional understanding, empathy, self-awareness, and self-reflection (33). For peer support workers to be able to undertake this important work it is essential that they are provided with necessary support within the organization.

Supervision guidelines have been developed for non-clinical settings, but there is still relatively little information on the types of supervision support needed by PSWs within a clinical setting (24). The diaries however suggest that to be able to work effectively within a community-based rehabilitation setting, it is critical to maintain the lived/living experience aspect of the roles. This can be achieved by regular peer supervision around their roles. In the HHS where this study was undertaken, the peer support workers, in addition to being supervised by their team leaders, also report to the Director of Social Inclusion and receive supervision from the lived experience workforce. This structure is intended to mitigate against some of the issues raised in the literature to date of perceived power imbalance of peers with clinicians, of aligning too closely with clinicians and losing their peer identity. Results from this study indicate that supervision is needed to guide the peer workers to not only maintain a lived experience focus, but also to work within the scope and the boundaries of their role. For the role of a peer support worker to be effective, it is important that this role is equally valued and understood by the team and the organization at large. Consequently, implementing peer roles within clinical settings necessitates a whole of workplace approach with a focus on organizational culture and supervision in effectively integrating peer workers within the service (20).

## Limitations

The diaries were written from the perspective of peer support workers and it is not possible to determine if residents experienced these interactions in the way that they were described. It is also important to note that peer support workers work within a community-based residential rehabilitation setting and that the challenges faced are likely to be very different to peer workers in a more acute and or shorter-term clinical environments. One of the major drawbacks of using a diary approach has been that the research team was not able to ask people to elaborate on different points and to clarify confusing statements. Peer support workers also emphasized the more positive aspect of their work. At the time when the data was collected, peer support workers were employed on a contract basis and the uncertainty about the stability of employment could have led to the peer support workers to emphasize the more positive aspects of their work. Since that time, however, the positions are now permanent. Despite these limitations, however, the insights gained from these diaries have provided valuable insights into how peer support workers conceptualize their practice and provided peer support workers with the opportunity to capture the experiences when they occurred.

## Conclusions

The findings of this study reinforce the value and unique contributions peer support workers can make, not only to the recovery journey of residents but also to the clinical team. Peer support workers described their work as flexible, responsive, and adaptable to the resident's needs. By sharing their experiences peer support workers were able to bring a different lens to situations and work inclusively with people. To ensure that peer workers can continue to provide this support it is critical that the peer support workers are supported through professional supervision and that the role and principles of peer work are understood, and peer workers embedded within the organization. This supervision could be enhanced by using reflective diaries which are an effective way for peer support workers to capture their own understanding and share the uniqueness and effectiveness of their work.

## Data Availability Statement

The datasets presented in this article are leave this in available because the data is confidential.

## Ethics Statement

The studies involving human participants were reviewed and approved by Metro South Health Ethics Committee.

## Author Contributions

MW and HR: design. GV, FD, and KM: implementation. MW, HR, GV, KM, SP, CE, and FD: data analysis and write up. All authors contributed to the article and approved the submitted version.

## Conflict of Interest

The authors declare that the research was conducted in the absence of any commercial or financial relationships that could be construed as a potential conflict of interest.
